# The highly expressed COL4A1 genes contributes to the proliferation and migration of the invasive ductal carcinomas

**DOI:** 10.18632/oncotarget.17345

**Published:** 2017-04-21

**Authors:** Rongzhong Jin, Jia Shen, Tiancheng Zhang, Qiliang Liu, Caihua Liao, Hailin Ma, Sijing Li, Zhaoxia Yu

**Affiliations:** ^1^ College of Biotechnology, Guilin Medical University, Guilin, China; ^2^ Tumor Initiation and Maintenance Program, Cancer Center, Sanford Burnham Prebys Medical Discovery Institute, La Jolla, California, USA; ^3^ Institute of Reproduction and Development, Fudan University, Shanghai, China; ^4^ China National Population and Family Planning Key Laboratory of Contraceptive Drugs and Devices, Shanghai Institute of Planned Parenthood Research (SIPPR), Shanghai, China; ^5^ Department of Medical Oncology, Central Hospital of Weihai, Weihai, Shandong, China

**Keywords:** IDC, biomarker, bioinformation, VCAN, COL4A1

## Abstract

**Background:**

Invasive ductal carcinoma is a kind of very typical breast cancer. The goal of our research was to figure out the molecular mechanism of Invasive ductal carcinoma and to find out its potential therapy targets.

**Results:**

The total amount of 478 differentially expressed genes in Invasive ductal carcinoma which compared with normal breast epithelial cells were recognized. Functional enrichment analysis proved the most part of differentially expressed genes had connection with ECM-receptor interaction. The two genes lists were contrasted in PPI network, and miRNA regulation networks, The most two crucial genes were identified in our study, which may be helpful to improve Invasive ductal carcinoma treatment. Additionally, experimental results shows that the COL4A1 gene, one of identified genes, played important roles in both of proliferation and colony formation in Invasive ductal carcinoma.

**Conclusions:**

Invasive ductal carcinoma could have connection with ECM-receptor mutations. These 9 vital genes could be an important part in the progression of Invasive ductal carcinoma and be offered as therapy targets and prognosis indicator. and the experimental results showed that one of the most crucial genes, COL4A1, was the key gene that influence the proliferation and colony formation of the Invasive ductal carcinoma cell.

## INTRODUCTION

Invasive ductal carcinomas (IDC) is a very typical breast cancer [1]. How to describe ductal carcinomas into a terminological phrase is still a controversial problem as on purely anatomy because of lacking of a wide-acceptable classification. IDC and some other types of breast cancer, like invasive lobular carcinomas (ILC) are generated from the terminal duct lobular unit (TDLU), and their morphologic distinction is impossible to show physiological source of lesions but the different parts in mechanisms of carcinogenesis. Both tumor types own a same statement in clinical pathological parameter as size, stage, etc [2], but researchers recognized that their pattern of advancement and further development are different which is based on new clinical data and analysis of pattern of metastasis [3, 4]. Matched-treatment is similar for tumors in different stage [5], but ILCs can always resist to treatment [6]. Compared with IDCs patients, ILCs Patients speared in older age stage and have low grade tumor and less lymphatic invasion, but their survival chance are similar [7, 8].

There is a useful technology, microarrays. It makes the synchronous study to the expression of enormous genes is possible. This technology is a combination of several functions, including tumor classification, molecular pathway modeling, operational genomics, and comparison of gene expression profiles between groups [9]. Ductal breast cancer have been classified into two classes, offensive phenotype and non-offensive phenotype by early researchers with microarrays. There are also some researchers identified that expression patterns is clear between BRCA1/2 status [10–13]. The gene expression in breast cancer was studied by many researchers by using microarray, but the carcinogenesis and pathophysiological mechanism of IDC isn't completely understood.

COL4A1, as the one kinds of the collagens which were the unique collagen constructed the basement membrances [14]. so the COL4A1 was tightly associated with the cell proliferation, it was reported that COL4A1 knockdown led to reduced cell viability and cell cycle arrest [15], and clinical performance associated with mutations of COL4A1 include perinatal cerebral hemorrhage and porencephaly [16], hereditary angiopathy, nephropathy, aneurysms, and muscle cramps (HANAC) [17], ocular dysgenesis, myopathy and Walker-Warburg syndrome [18]. the latest reports identify the truncation of C-terminal NC1 domain of type IV collagen 1 by frameshift mutation tightly linked with renal disease and demonstrate that the highly conserved C-terminal part of the NC1 domain of the α1 chain of type IV collagen is important in the integrity of glomerular basement membrane in humans [19].

In this research, we collected 15 microarray data from microarray dataset: normal ductal cells from 10 patients; five surgical specimens with IDC and proceeded several of bioinformatics analyses to recognize the molecular mechanism of IDC [20]. We identified the different expressions between normal cells and cancer cells. Several genes that might have a crucial function in the development of IDC were also founded. based on the bioinformatics analysis, a series of the experiments were performed to verify the results obtained from the data mining, and indicated that overexpressed COL4A1 gene promote the proliferation of the invasive ductal carcinomas cells SKBR3.

## RESULTS

### Analysis of DEGs

**Figure 1:**
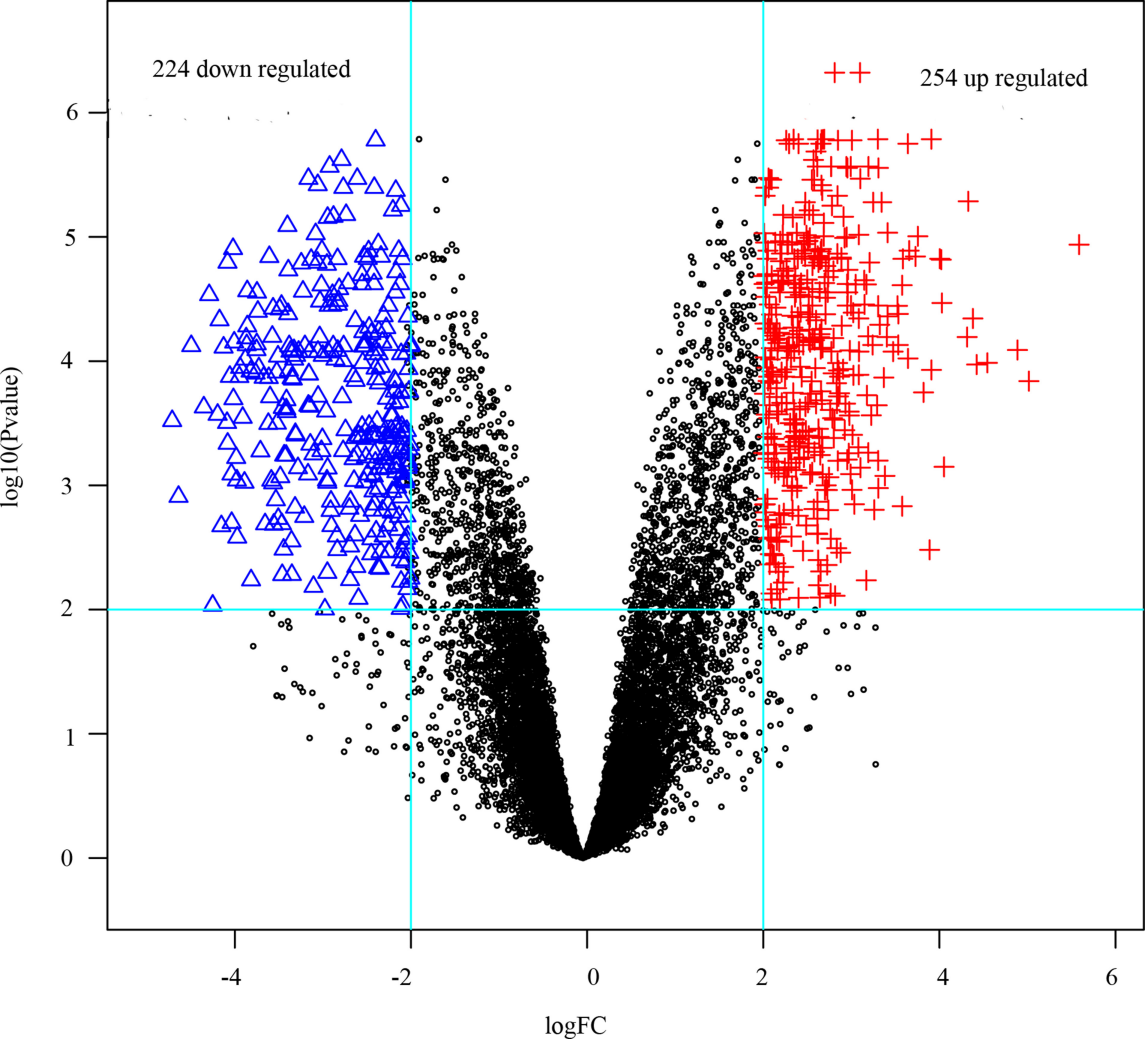
The DEGs in ductal tumor samples compared with those in normal samples Blue triangles and red plus signs represented down- and up-regulated genes respectively, black dots were non-differentially expressed genes.

By examining microarray data in normal ductal cells with IDC, a sum amount of 478 DEGs (1.5% of total) were recognized. There are 254 up-regulated genes and 224 down-regulated ones (Figure 1). The most ten up-regualted genes were TFPI2, ARHGAP36, THSD7B, ATP1A2, KRT14, SCGB1D2, MUCL1, KRT5, KRT16 and PPP4R4. The most ten down-regualted genes were CLEC7A, TOP2A, COL1A2, CLEC2B, ANGPT2, TCF4, MLLT10, COL11A1, FAR2 and SMEK1 (Table 1).

**Table 1:** The top 10 most up and down regulated genes

Up regulated			Down regulated		
Gene Symbol	logFC	*P* value	Gene Symbol	logFC	*P* value
TFPI2	−5.60	2.63 × 10^−3^	CLEC7A	4.50	2.76 × 10^−4^
ARHGAP36	−5.26	3.34 × 10^−3^	TOP2A	4.17	1.33 × 10^−3^
THSD7B	−5.07	2.99 × 10^−5^	COL1A2	4.04	1.89 × 10^−3^
ATP1A2	−5.04	2.41 × 10^−6^	CLEC2B	4.04	1.90 × 10^−3^
KRT14	−4.98	2.55 × 10^−5^	ANGPT2	3.99	8.00 × 10^−3^
SCGB1D2	−4.97	1.87 × 10^−3^	TCF4	3.97	6.50 × 10^−3^
MUCL1	−4.91	5.97 × 10^−3^	MLLT10	3.90	5.53 × 10^−3^
KRT5	−4.84	1.30 × 10^−4^	COL11A1	3.89	1.50 × 10^−3^
KRT16	−4.78	9.88 × 10^−5^	FAR2	3.68	2.27 × 10^−3^
PPP4R4	−4.64	2.13 × 10^−3^	SMEK1	3.60	2.10 × 10^−3^

### Function and pathway enrichment analysis

**Figure 2:**
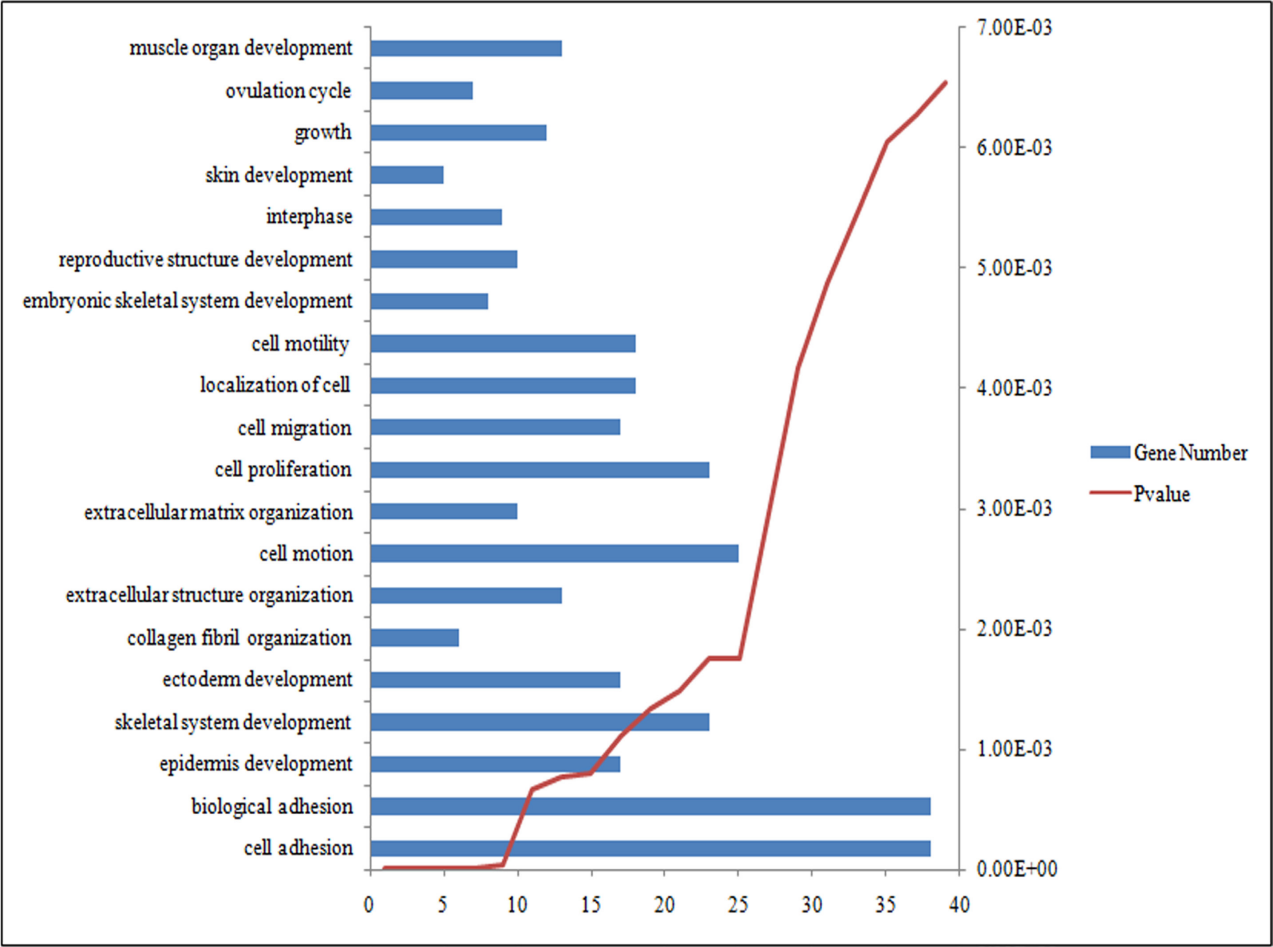
The top 20 most significantly enriched GO terms of DEGs according *P* value

478 DEGs in gene list was uploaded to DAVID internet site and conducted GO analysis which *p* under or equal to 0.05. In Figure 2 showed the top 10 enriched GO terms. We found that most of them were connected with cytoplasmic movement method (6/10), including Cell adhesion (20.5% DEGs were enriched with *p* = 3.64E-10), biological adhesion (15.66%; *p* = 1.18E-06), skeletal system development (12.05%; *p* = 4.79E-06), extracellular structure organization (12.05%; *p* = 2.3E-05) and cell motion. Not only cytoplasmic movement system, but GO terms connected with cellular development were also seemed to being triggered, such as spidermis development (15.66%; *p* = 1.73E-08), cell proliferation (10.84%; *p* = 1.62E-05). KEGG pathway analysis showed a same results (Table 2), with one of the considerable KEGG term of ECM-receptor interaction (9.64%; *p* = 2.86E-05). Plus, Focal adhesion (8.43%; *p* = 6.99E-04), p53 signaling pathway (8.43%; *p* = 0.0015), Cell cycle(9.64%; *p* = 2.86E-05). In the total 9 KEGG pathways and 10 GO enriched terms, there were 15 flapped DEGs which could be connected to IDC: CXCL13, LCP2, VAV1, PTPRC, FCGR2B, FCGR3A, CX3CR1, PYCARD, IL18, RAC2, IFI30, INPP5D, CCL2, PTPN6, and STAT3.

**Table 2:** The enriched pathways of the DEGs

Pathway Name	*P* value	Gene Number
hsa04512:ECM-receptor interaction	6.62 × 10^−7^	13
hsa04510:Focal adhesion	9.40 × 10^−5^	16
hsa04115:p53 signaling pathway	0.0244	6
hsa04110:Cell cycle	0.0448	7

### Analysis of PPI network

**Figure 3:**
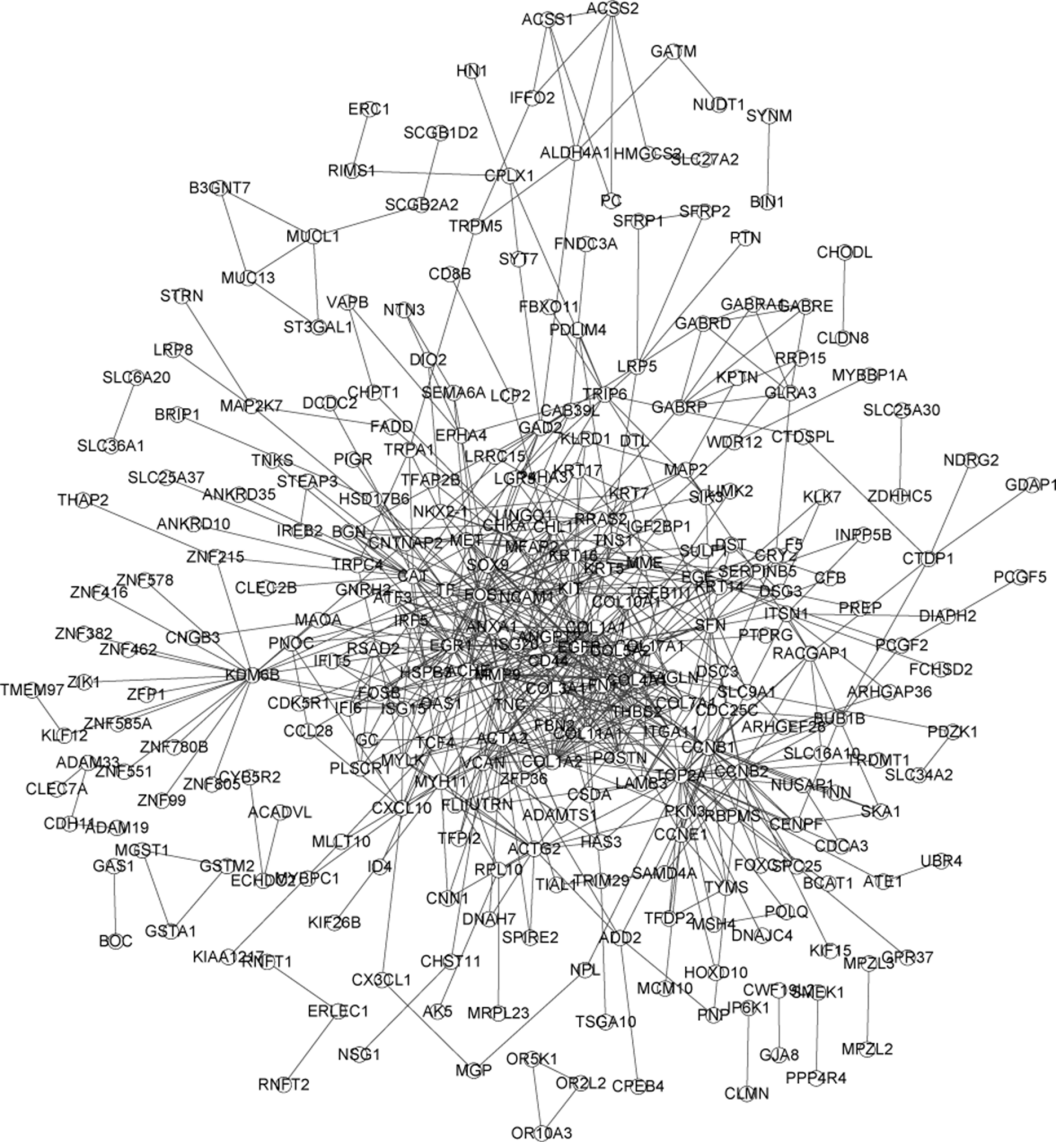
PPI network of DEGs obtained from the STRING database

Initially, to get PPI data, we uploaded 478 DEGs to STRING website. Next, the samples whose PPIs data over 0.4 were picked to assemble PPI networks. STRING extracted 562 pairs of PPI which were containing 41 DEGs. These genes were generally allocated in one PPI image (Figure 3) to find out their connections,. The top 20 degree hub nodes were thus: RAC2, CD4, CD68, PTPRC, CCL2, IL18, PTPN6, ARPC1B, STAT3, CD48,LCP2, CX3CR1, FCGR3A, IFI30, INPP5D, PLEK, PYCARD, ARHGDIB, CORO1A, and CYBA. These genes (proteins) could be a crucial part in the IDC development.

### Analysis of Mi-RNA/gene network

**Figure 4:**
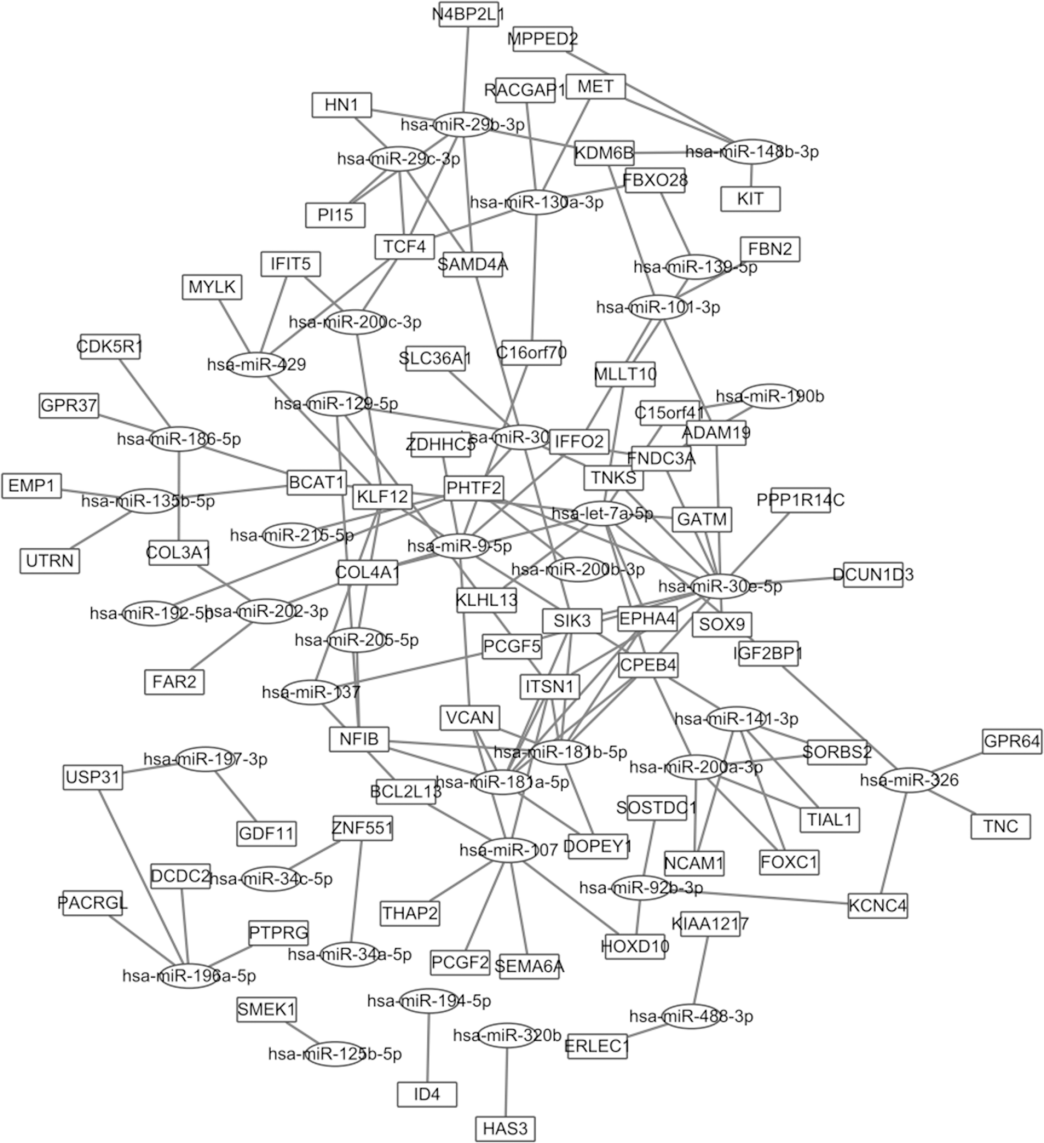
miRNA-gene network of DEGs obtained from the starBase database

Based on the starBase, a total of 37 potential regulation MiRNA and 125 MiRNA-Gene regulation pairs were obtained (Figure 4). The top 20 according to their node degree of co-expression network were PTPRC, MX1, FCGR3A, UGT1A6, LY86, GRN, CP, TSPO, CD68, SKAP2, CD53 Table 3. By comparing those two lists of DEGs in PPI network, and MiRNA-Gene regulation network, we found that, 9 genes existed in MiRNA-Gene regulation network regulated by at least 3 miRNAs and interacted with at least 3 genes in PPI network: ITSN1, TCF4, EPHA4, VCAN, SIK3, IFFO2, SAMD4A, KMM6B and COL4A1.These overlapped DEGs could be more crucial part in IDC.

**Table 3:** The genes that regulated by at least 3 miRNAs in miRNA-gene regulation network and have at least 3 direct neighbors in PPI network

Gene Symbol	miRNAs	Neighbors
ITSN1	5	8
TCF4	5	6
EPHA4	5	6
VCAN	4	14
SIK3	4	3
IFFO2	4	3
SAMD4A	3	3
KMM6B	3	20
COL4A1	3	16

### Key genes filter and survival analysis

**Figure 5:**
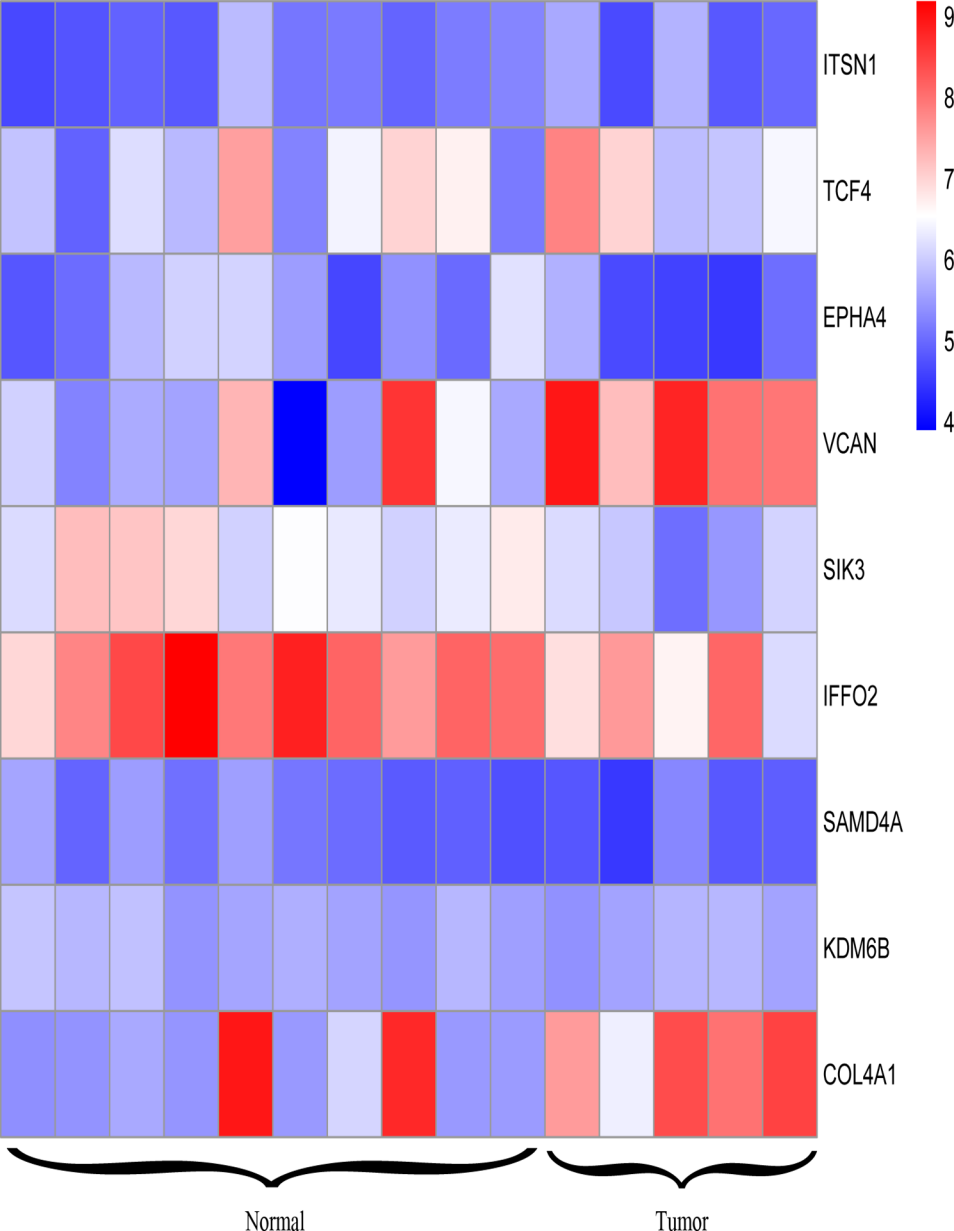
Heatmap of genes that have at least 3 neighbors in PPI network as well as miRNA-gene regulation network

**Figure 6:**
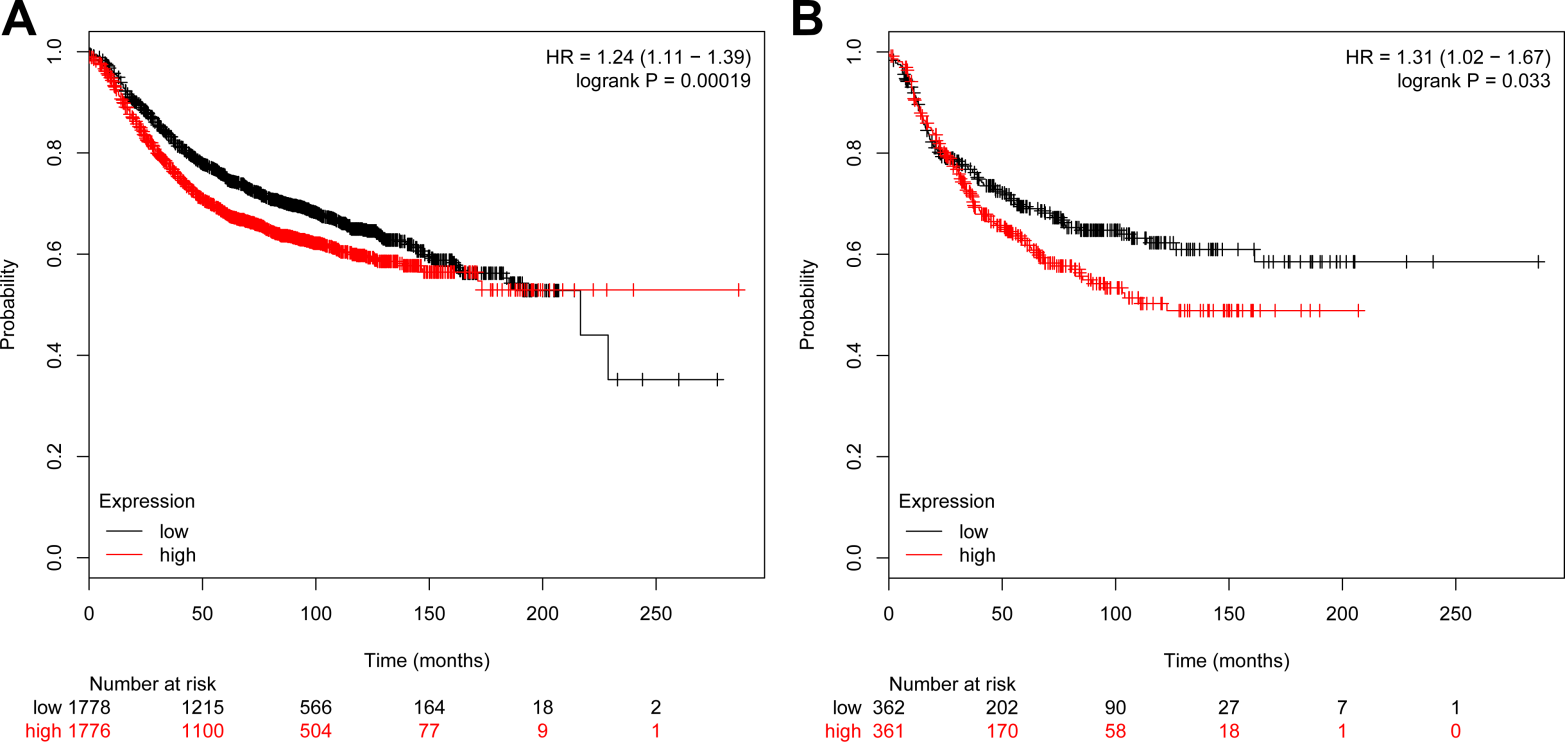
Survival curves of ductal tumor patients associated with VCAN and COL4A1 **(A)** represented the survival curve of COL4A1 and **(B)** represented the survival curve of VCAN.

To visualizing gene expression level of the 9 most overlapped genes, we used pheatmap package implemented in R to generate a heatmap (Figure 5) to detect the gene expression differences between normal ductal cells and IDC. In this step, we targeted two genes: VCAN and COL4A1,because they showed the most distinction in gene expression profile. To check out if the expression status of VCAN and COL4A1 has any medical value for treatment, we did analyses by using the Kaplan–Meier method. As data displayed in Figure 6A and Figure 6B, lower expression of VCAN or COL4A1 predicted a longer overall survival in patients (*P* = 0.007; *P* < 0.001).

### The mRNA level of COL4A1 were up-regulated in the SKBR3 cells

**Figure 7:**
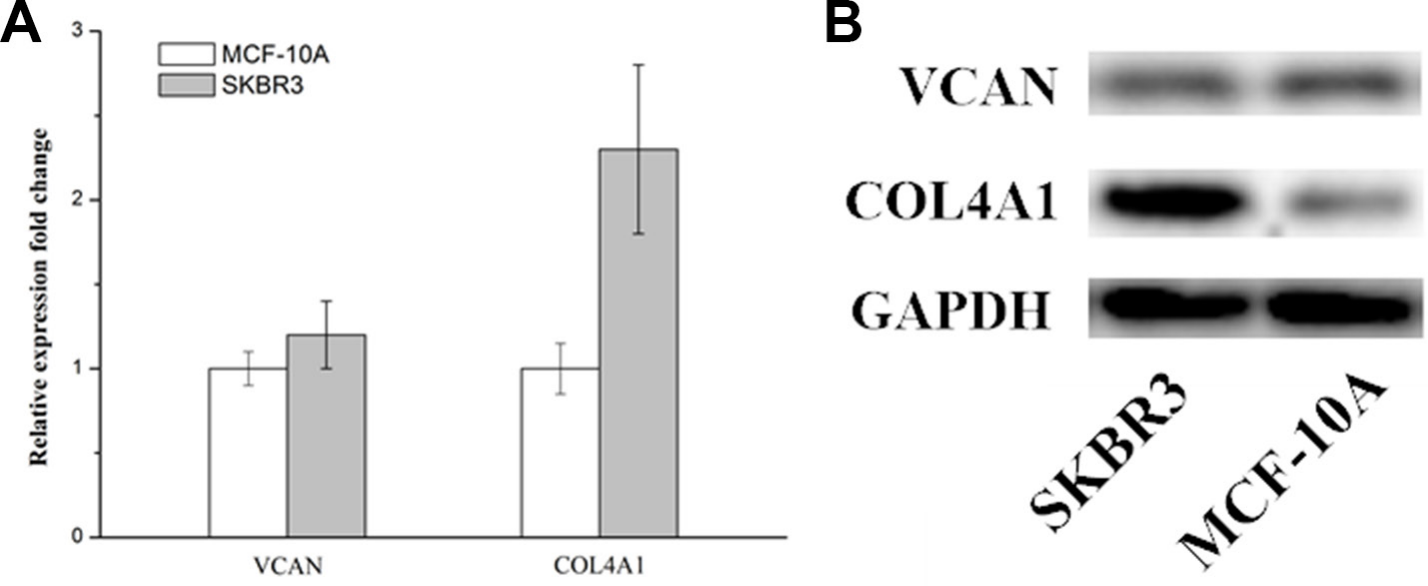
Versican and COL4A1 mRNA levels in SKBR3 cells (**A**) Quantitative PCR analysis. Relative mRNA expression levels were calculated as a ratio of mRNA levels of the genes of interest to those of Control (BEAS-2B) (**B**) Relative protein expression levels were evaluated by western blot.

In order to assess the expression of candidate genes in the SKBR3 cells, RT-PCR and Western Blot was employed and the results were shown in Figure 7A and 7B, compared to the control (the normal lung epithelial cells BEAS-2B), a up-regulation in the mRNA levels of the gene COL4A1 was observed which is consistent with the previous findings from bioinformatics analysis. but for the VCAN gene. no significant change in the mRNA level were observed compared with the control. these results showed that COL4A1 gene were up-regulated in the SKBR3 cells.

### Knocking down COL4A1 gene in SKBR3 cells

**Figure 8:**
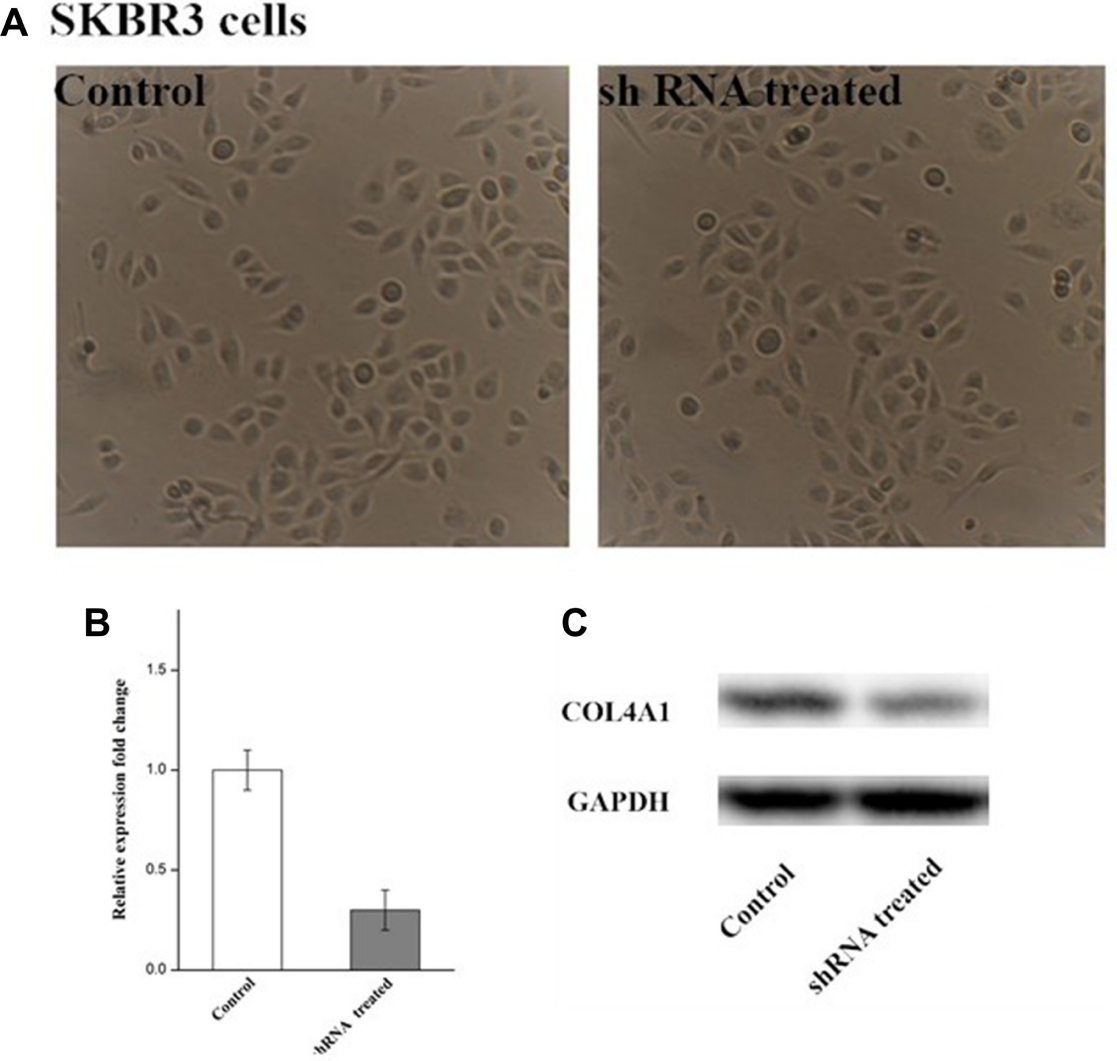
No obvious change in the cell morphology in the shRNA treated group, as compared with the control (**A**) and the expressions of COL4A1 gene and protein were measure by RT-PCR (**B**) and Western blot (**C**) respectively.

Lentiviral vector delivery of shRNA targeting the Col4A1 gene into the SKBR3 cells resulted in the reduction of COL4A1 mRNA and protein, as measured by RT-PCR and Western blot analysis, respectively. as can be seen from the Figure 8, the corresponding expression of COL4A1 in the mRNA and protein levels were obviously down-regulated by shRNA treatment. andthe cell treated with COL4A1 shRNA did not show obvious difference in morphology, indicating no significant change in cytoskeletal architecture by the shRNA treatment.

### Knocking down the COL4A1 gene significantly reduced the proliferation and colony formation

**Figure 9:**
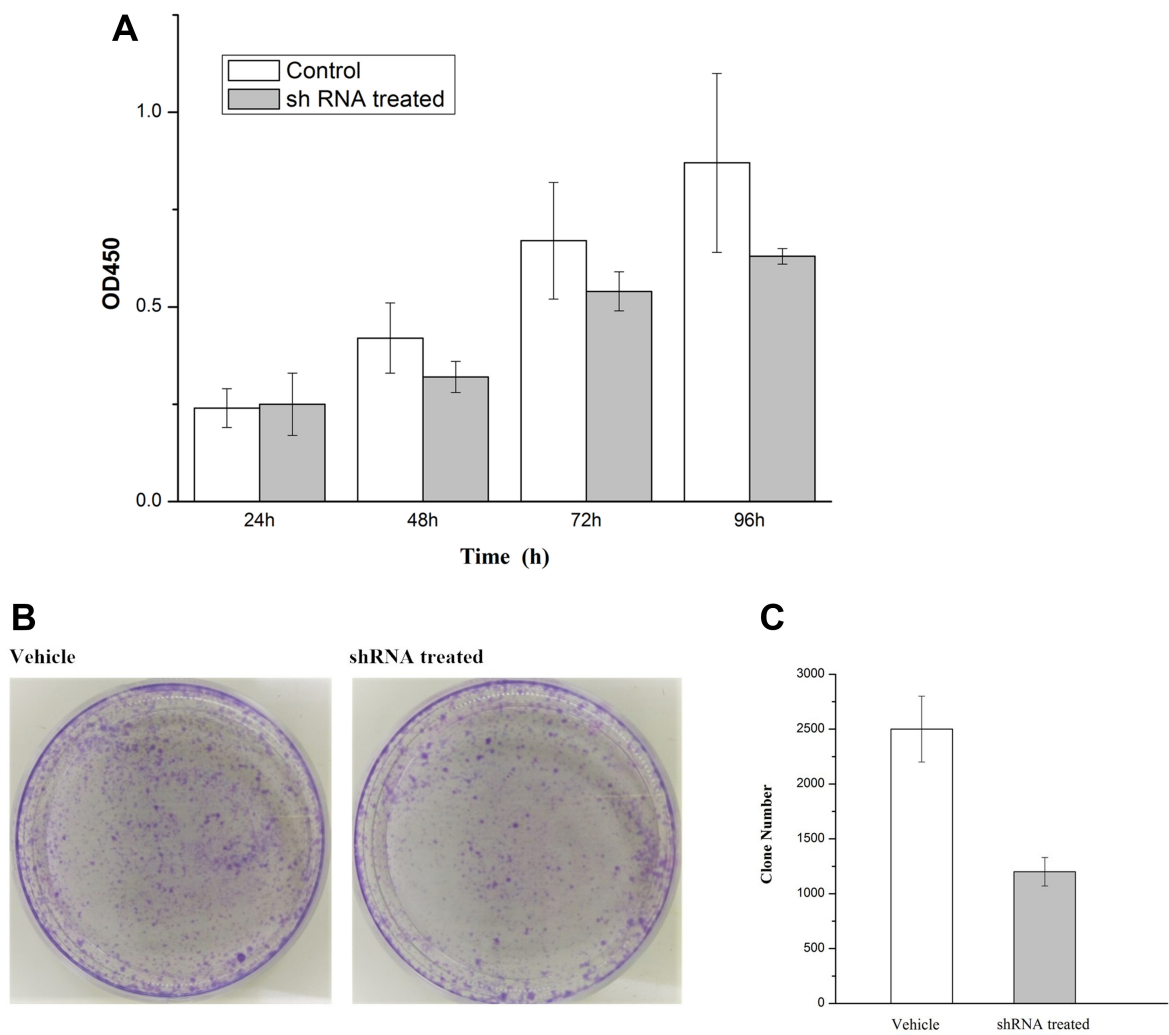
(**A**) Knockdown of COL4A1 greatly inhibited proliferate ability of SKBR3 in 96 h. (**B**–**C**) Photomicrographs of Giemsa-statined colonies of SKBR3 cells growing in 6-well plates for 5 days after infection the number of cells in each colony was counted. Cell number in shRNA treated was significantly reduced as compared with vehicle group (*p* < 0.001).

To assess the roles of COL4A1 in regulating IDC cell proliferation. the SKBR3 cells were infected by COL4A1 shRNA or control shRNA lentivirus. all treated cells were counted continuously for 4 days by spectroscopic assay. SKBR3 cells proliferation were inhibited significantly by COL4A1 knockdown (Figure 9, Upper). To explore the effect of COL4A1 in IDC colony formation ability. SKBR3 cells treated with COL4A1 shRNA or control shRNA lentivirus were allowed to grow for 14 days to form colonies. as shown in Figure 9, Lower. COL4A1 knockout resulted in significantly decrease in the number of colonies in SKBR3 cells, as compared with the control shRNA group. Thus, our data indicated that the COL4A1 is able to regulate colony forming ability of IDC.

## DISCUSSION

IDC also called infiltrating ductal breast carcinoma, is a frequent style, covering mostly 70–80% of all breast cancer diagnoses. IDC invades man with the highest rate of frequency [21]. Study on DEGs between IDC and normal ductal cells might be helpful in finding related genes of invasive ductal breast carcinoma. In this research, we collected the Microarray data of GSE5764 from GEO system, and 478 DEGs particular expression in IDC were identified. We speculated that they were possible to have a connenction with invasive ductal breast carcinoma. GO and KEGG enrichment analysis, PPI network analysis, MiRNA-Gene interatction network analysis were done and resulted the 478 DEGs. We found there were exciting consequences. With the help from GO enrichment analysis, there seems to be a clear connection between the DEGs and GO terms of cytoplasmic movement. KEGG pathway analysis also proved that.

Besides, in this research, we recognized 9 genes flapped in both gene lists. They associate with ECM-receptor interaction. Two of them, VCAN and COL4A1, show the most different expression profile between IDC and normal ductal cells. VCAN was called as proteoglycan-M (PG-M), is a large hyaluronan (HA)-binding chondroitin sulfate extracellular matrix proteoglycan which is part of the lectican family [22]. but experimental results showed that COL4A1 was significant upregulated gene compared with the control, but VCAN was not obvious upregulated in the SKBR3 cell line, COL4A1 encode type IV collagen was found in nearly all basement membranes and is truly preserved within species, and comprise 52 and 48 exons specifically. They can be divided into 127 nucleotides containing a shared, bi-directional promoter that requires alternative factors to control the structure uniqueness and the level of protein expression [23]. Herein, we described that COL4A1 is a negative prognosis symbol in invasive ductal breast cancer. This result is consistent with a former research, which had reported that collagen IV expressed by COL4A1 can be regulated by P4HA2 in tumor growth and metastasis [24]. and also the COL4A1 was also identified as the potential therapeutic target genes in head and neck squamous cell carcinoma [25], colorectal carcinoma [26] and thyroid papillary carcinoma [27], it was also found that COL4A1 was the most significantly upregulated genes during the formation of the avian blood-gas barrier. Mutations in COL4A1 derived from the vascular component were sufficient to cause defects in vascular development and the blood-gas barrier. and mutation in COL4A1 resulted in disrupted myofibroblast proliferation, differentiation and migration [28]. where this result are also consisted with our experimental results that COL4A1 silence lead to the suppress in proliferation, differentiation and migration of IDC.

## MATERIALS AND METHODS

### bioinformation data mining

### Microarray data

The microarray data GSE5764 was collected from Gene Expression Omnibus(GEO) which built upon the platform of Affymetrix Human Genome U133 Plus 2.0 Array. This platform was stored by Turashvili *et al*. [20] which included 30 microarray data of 10 patients’ regular ductal and lobular cells, 10 surgical samples which were acquired by mastectomy from postmenopausal patients with IDC and ILC were checked out. There were 5 IDCs and 5 ILCs.

### Data preprocessing

The original CEL data were imported into R and affy package was used for the background correction and normalization. The expression of genes that corresponding to multi probes were summaried. By the function of mas5calls, those samples which had no gene express were taken out in Affy package. Finally, we obtained 8642 genes in expression levels.

### Differentially expressed genes selection

There are four kinds of samples in the datase, 10 normal ductal cells, 10 normal lobular cells, 5 IDC and 5 ILC. DEGs between 10 normal ductal cells and 5 IDC were identified with the use of Limma package [29]. The *p*-value under or equal to 0.05 and |log2fold change| larger than 1 were decided as check standard.

### Functional annotation and pathway analysis of DEGs

Database for Annotation, Visualization, and Integrated Discovery [30] is a online program that combines functional genomic annotations with intuitive graphical summaries [31]. Gene lists or protein identifiers are swiftly annotated and concluded with the using of comprehensive categorical data for Gene Ontology (GO), protein domain, and biochemical pathway membership. For making a extensive evaluation of connected pathway or biologic methods in IDC, GO and Pathway enrichment analysis on DEGs were executed with the DAVID analysis system with threshold of *p*-value under or equal to 0.05.

### Protein interaction networks analysis

Retrieval of Interacting Genes/Proteins (STRING) [32] database (http://string-db.org/) was decided to be the Search Tool to provides a chance to reach all interaction data, including data which might be unsafe and/or predictions made by computers. There are more than 1100 organisms in extensive protein connection with global data. In this research, protein-protein interaction (PPI) network of DEGs were founded by STRING online database with threshold of score lager than 0.4. The hub protein was selected by the node degree, and the network were visualized by Cytoscape [33].

### MiRNA-gene regulation network analysis

The starBase (http://starbase.sysu.edu.cn/) is a database that deciphers the regulation relationships between RNA and RNA, or protein and RNA by managing the 108 CLIP-Seq datasets from 37 independent studies [34]. It can provide miRNA-mRNA regulation pairs when using a different parameter setting. In this research, miRNA-mRNA interaction network of DEGs were collected by starBase online database.

### Kaplan–Meier survival analysis

KM Plotter (http://kmplot.com/analysis/) is used for the meta-analysis based biomarker analysis in this paper [35] which analysed more than thousands of gene expression samples of breast cancer patients, was used to work out Kaplan–Meier survival evaluation to further assess the relationship of DEGs and prognosis. In the survival evaluation quartiles were used as cutoff values and it was set at p under 0.05.

### cells and reagents

a human breast cancer cell line (SKBR3) and normal lung epithelial cell line (BEAS-2B) were obtained from the ATCC and cultured in low-glucose Dullbecco's modified eagle's medium (DMEM),

### Real-time PCR

Total RNA was extracted from the cultured MCF7 cells and the concentration of extracted RNA was estimated by optical density measurement (A260/A280 ratio) with a NanoVus Plus (GE, USA). Real-time PCR amplification was carried out using the SYBR Green-based PCR Master Mix (Applied Biosystems/Life Technologies, USA). The ABI PRISM 7500 system (ABI, USA) was used for all reactions in a total volume of 25 μL.

### Western blotting

Cells were harvested and total protein was isolated using RIPA buffer (50 mM Tris-HCl at pH 7.4, 1 mM EDTA, 150 mM NaCl, 1% NP-40 and 0.5% SDS) supplemented with proteinase cocktail (Roche, Switzerland), and heated for 5 min at 100°C. BCA Protein Assay Kit (Takara, Japan) was used to determine protein concentration. Equal amount of denatured protein samples were loaded and separated by 10% SDS-polyacrylamide gels, and then transferred onto polyvinylidene difluoride membranes (PALL). After blocking with 5% non-fat milk power in Tris-buffered saline/0.05% Tween 20(TBST), the membrane was incubated with a specific primary antibody, followed by the HRP-conjugated secondary antibody. Proteins were visualized using ECL reagents (Tanon, China).

### Lentivirus production and cell transduction

To knock-down the expression of COL4A1, SKBR3 cells were transducted with lentivirus carrying shRNA (sequence, CCGGCCTGGGATTG ATGGAGTTAAACTCGAGTTTAACTCCATCAATCC CAGGTTTTTG). To produce lentivirus, 15 ug of the transfer plasmid, 9ug of the package plasmid (psPAX2), and 6 ug of the envelope plasmid (pMD2.G) were cotransfected into 293T cells by the calcium phosphate method. After 24 h of transfection, the cells were cultured in 15 ml of fresh serum-free medium for another 48 h. The culture medium with virions was then collected, filtered through a 0.45-um filter, and centrifuged at 90,000 g for 90 min. For transduction, SKBR3 cells were infected for 16 h with 10 ul of virus suspension containing 8 ug/ml of Polybrene (Sigma)

### Cell proliferation

Cell proliferation was assessed by water-soluble tetrazolium salt (WST) assay with the Cell Counting Kit-8 (Dojindo, Kumamoto, Japan) and measured per the manufacturer's instructions. At 24 hours after transfection with vehicle or COL4A1 shRNA, SKBR3 cells were seeded onto 96-well plates (2 × 10^3^ cells/well), and cell proliferation was documented every 24 hours for 4 days. The number of viable cells was assessed by measurement of the absorbance at 450 nm.

### Colony-formation assay

In plate colony-formation assay, malignant melanoma cells were resuspended in RPMI 1640 containing 10% FBS and layered onto 6-well plates (5 × 10^2^ cells/well). The cells were incubated for 5 days and stained with Giemsa. Colonies containing 50 cells or more were counted.

### Statistical analysis

Data are shown as the means ± standard deviation. The statistical significance of differences between groups was assessed via one-way analysis followed by Student's *t*-tests of comparison. *p*-values less than 0.05 were considered to be statistically significant. Statistical analyses were conducted using the GraphPad Prism 4.0 software.

## CONCLUSIONS

In this research, a general amount of 478 DEGs were identified to have a connection with pathological IDC. Within them, 9 vital genes were additionally recognized by equating PPI network, and MiRNA-Gene network. Two of them were selected to evaluate the relationship between their expression and prognosis. moreover, experimental results indicated that down-regulated expression of COL4A1 gene significantly inhibit the colony formation of the IDC cells and suppress the proliferation ability of the IDC cells. These genes could be a crucial part in the progression of IDC and be offered as therapy targets and prognosis indicator.

## Abbreviations

IDC: Invasive ductal breast cancer
GEO: Gene Expression Omnibus
DEGs: Differentially expressed genes
GO: DAVID: Gene Ontology
PPI: Protein-protein interaction
ILC: invasive lobular carcinomas
TDLU: terminal duct lobular unit
STRING: The Search Tool for the Retrieval of Interacting Genes/Proteins.

## References

[1] Weigelt B, Geyer FC, Reis-Filho JS (2010). Histological types of breast cancer: how special are they?. Mol Oncol.

[2] Winchester DJ, Chang HR, Graves TA, Menck HR, Bland KI, Winchester DP (1998). A comparative analysis of lobular and ductal carcinoma of the breast: presentation, treatment, and outcomes. J Am Coll Surg.

[3] Silverstein MJ, Lewinsky BS, Waisman JR, Gierson ED, Colburn WJ, Senofsky GM, Gamagami P (1994). Infiltrating lobular carcinoma. Is it different from infiltrating duct carcinoma?. Cancer.

[4] Toikkanen S, Pylkkänen L, Joensuu H (1997). Invasive lobular carcinoma of the breast has better short- and long-term survival than invasive ductal carcinoma. Br J Cancer.

[5] Molland JG, Donnellan M, Janu NC, Carmalt HL, Kennedy CW, Gillett DJ (2004). Infiltrating lobular carcinoma—a comparison of diagnosis, management and outcome with infiltrating duct carcinoma. Breast.

[6] Newman LA, Buzdar AU, Singletary SE, Kuerer HM, Buchholz T, Ames FC, Ross MI, Hunt KK (2002). A prospective trial of preoperative chemotherapy in resectable breast cancer: predictors of breast-conservation therapy feasibility. Ann Surg Oncol.

[7] Mersin H, Yildirim E, Gülben K, Berberoğlu U (2003). Is invasive lobular carcinoma different from invasive ductal carcinoma?. Eur J Surg Oncol.

[8] Arpino G, Bardou VJ, Clark GM, Elledge RM (2004). Infiltrating lobular carcinoma of the breast: tumor characteristics and clinical outcome. Breast Cancer Res.

[9] Bertucci F, Viens P, Hingamp P, Nasser V, Houlgatte R, Birnbaum D (2003). Breast cancer revisited using DNA array-based gene expression profiling. Int J Cancer.

[10] Sørlie T, Perou CM, Tibshirani R, Aas T, Geisler S, Johnsen H, Hastie T, Eisen MB, van de Rijn M, Jeffrey SS, Thorsen T, Quist H, Matese JC (2001). Gene expression patterns of breast carcinomas distinguish tumor subclasses with clinical implications. Proc Natl Acad Sci USA.

[11] van de Rijn M, Perou CM, Tibshirani R, Haas P, Kallioniemi O, Kononen J, Torhorst J, Sauter G, Zuber M, Köchli OR, Mross F, Dieterich H, Seitz R (2002). Expression of cytokeratins 17 and 5 identifies a group of breast carcinomas with poor clinical outcome. Am J Pathol.

[12] van 't Veer LJ, Dai H, van de Vijver MJ, He YD, Hart AA, Bernards R, Friend SH (2003). Expression profiling predicts outcome in breast cancer. Breast Cancer Res.

[13] Hedenfalk I, Duggan D, Chen Y, Radmacher M, Bittner M, Simon R, Meltzer P, Gusterson B, Esteller M, Kallioniemi OP, Wilfond B, Borg A, Trent J (2001). Gene-expression profiles in hereditary breast cancer. N Engl J Med.

[14] Kühn K (1995). Basement membrane (type IV) collagen. Matrix Biol.

[15] Salem O, Erdem N, Jung J, Munstermann E, Wörner A, Wilhelm Heike, Wiemann Stefan, Cindy Körner (2016). The highly expressed 5 ‘ isomiR of hsa-miR-140–3p contributes to the tumor-suppressive effects of miR-140 by reducing breast cancer proliferation and migration. BMC Genomics.

[16] Gould DB, Phalan FC, Breedveld GJ, van Mil SE, Smith RS, Schimenti JC, Aguglia U, van der Knaap MS, Heutink P, John SW (2005). Mutations in Col4a1 cause perinatal cerebral hemorrhage and porencephaly. Science.

[17] Plaisier E, Gribouval O, Alamowitch S, Mougenot B, Prost C, Verpont MC, Marro B, Desmettre T, Cohen SY, Roullet E, Dracon M, Fardeau M, Van Agtmael T (2007). COL4A1 mutations and hereditary angiopathy, nephropathy, aneurysms, and muscle cramps. N Engl J Med.

[18] Labelle-Dumais C, Dilworth DJ, Harrington EP, de Leau M, Lyons D, Kabaeva Z, Manzini MC, Dobyns WB, Walsh CA, Michele DE, Gould DB (2011). COL4A1 mutations cause ocular dysgenesis, neuronal localization defects, and myopathy in mice and Walker-Warburg syndrome in humans. PLoS Genet.

[19] Gale DP, Oygar DD, Lin F, Oygar PD, Khan N, Connor TM, Lapsley M, Maxwell PH, Neild GH (2016). A novel COL4A1 frameshift mutation in familial kidney disease: the importance of the C-terminal NC1 domain of type IV collagen. Nephrol Dial Transplant.

[20] Turashvili G, Bouchal J, Baumforth K, Wei W, Dziechciarkova M, Ehrmann J, Klein J, Fridman E, Skarda J, Srovnal J, Hajduch M, Murray P, Kolar Z (2007). Novel markers for differentiation of lobular and ductal invasive breast carcinomas by laser microdissection and microarray analysis. BMC Cancer.

[21] Arps DP, Healy P, Zhao L, Kleer CG, Pang JC (2013). Invasive ductal carcinoma with lobular features: a comparison study to invasive ductal and invasive lobular carcinomas of the breast. Breast Cancer Res Treat.

[22] Kimata K, Oike Y, Tani K, Shinomura T, Yamagata M, Uritani M, Suzuki S (1986). A large chondroitin sulfate proteoglycan (PG-M) synthesized before chondrogenesis in the limb bud of chick embryo. J Biol Chem.

[23] Haniel A, Welge-Lüssen U, Kühn K, Pöschl E (1995). Identification and characterization of a novel transcriptional silencer in the human collagen type IV gene COL4A2. J Biol Chem.

[24] Xiong G, Deng L, Zhu J, Rychahou PG, Xu R (2014). Prolyl-4-hydroxylase α subunit 2 promotes breast cancer progression and metastasis by regulating collagen deposition. BMC Cancer.

[25] Kuang J, Zhao M, Li H, Dang W, Li W (2016). Identification of potential therapeutic target genes and mechanisms in head and neck squamous cell carcinoma by bioinformatics analysis. Oncol Lett.

[26] Qiao J, Fang CY, Chen SX, Wang XQ, Cui SJ, Liu XH, Jiang YH, Wang J, Zhang Y, Yang PY, Liu F (2015). Stroma derived COL6A3 is a potential prognosis marker of colorectal carcinoma revealed by quantitative proteomics. Oncotarget.

[27] Cong D, He M, Chen S, Liu X, Liu X, Sun H (2015). Expression profiles of pivotal microRNAs and targets in thyroid papillary carcinoma: an analysis of The Cancer Genome Atlas. Onco Targets Ther.

[28] Loscertales M, Nicolaou F, Jeanne M, Longoni M, Gould DB, Sun Y, Maalouf FI, Nagy N, Donahoe PK (2016). Type IV collagen drives alveolar epithelial-endothelial association and the morphogenetic movements of septation. BMC Biol.

[29] Diboun I, Wernisch L, Orengo CA, Koltzenburg M (2006). Microarray analysis after RNA amplification can detect pronounced differences in gene expression using limma. BMC Genomics.

[30] Carrera P, Di Resta C, Volonteri C, Castiglioni E, Bonfiglio S, Lazarevic D, Cittaro D, Stupka E, Ferrari M, Somaschini M, BPD and Genetics Study Group (2015). Exome sequencing and pathway analysis for identification of genetic variability relevant for bronchopulmonary dysplasia (BPD) in preterm newborns: A pilot study. Clinica Chimica Acta 451.

[31] Dennis G, Sherman BT, Hosack DA, Yang J, Gao W, Lane HC, Lempicki RA (2003). DAVID: Database for Annotation, Visualization, and Integrated Discovery. Genome Biol.

[32] Franceschini A, Szklarczyk D, Frankild S, Kuhn M, Simonovic M, Roth A, Lin J, Minguez P, Bork P, von Mering C, Jensen LJ (2013). STRING v9.1: protein-protein interaction networks, with increased coverage and integration. Nucleic Acids Res.

[33] Shannon P, Markiel A, Ozier O, Baliga NS, Wang JT, Ramage D, Amin N, Schwikowski B, Ideker T (2003). Cytoscape: a software environment for integrated models of biomolecular interaction networks. Genome Res.

[34] Li P, Sheng C, Huang L, Zhang H, Huang L, Cheng Z, Zhu Q (2014). MiR-183/-96/-182 cluster is up-regulated in most breast cancers and increases cell proliferation and migration. Breast Cancer Res.

[35] Győrffy B, Surowiak P, Budczies J, Lánczky A (2013). Online survival analysis software to assess the prognostic value of biomarkers using transcriptomic data in non-small-cell lung cancer. PLoS One.

